# Greater Cognitive Intraindividual Variability Predicts Faster Decline in Everyday Functioning: Moderating Effects of Amyloid Status and APOE Genotype

**DOI:** 10.1002/alz70857_101134

**Published:** 2025-12-25

**Authors:** Katherine J. Bangen, Lauren C. Edwards, Fini Chang, Fareshte Erani, Caitlin M. Terao, Kelsey R. Thomas

**Affiliations:** ^1^ University of California, San Diego, La Jolla, CA, USA; ^2^ VA San Diego Healthcare System, San Diego, CA, USA; ^3^ San Diego State University/University of California, San Diego Joint Doctoral Program in Clinical Psychology, San Diego, CA, USA; ^4^ San Diego State University/University of California San Diego Joint Doctoral Program in Clinical Psychology, San Diego, CA, USA

## Abstract

**Background:**

Cognitive intraindividual variability (IIV), or within‐person variability across cognitive measures at a single time point, is associated with increased risk of mild cognitive impairment (MCI) and Alzheimer's disease (AD) dementia. We have previously shown that IIV is a sensitive marker of future declines in everyday functioning, but little is known about its role as a sensitive marker of risk for decline among specific groups, including individuals who are amyloid‐β (Aβ) positive or apolipoprotein E (APOE) ε4 carriers. Therefore, we investigated potential moderating effects of Aβ status and APOE genotype on the association between IIV and everyday functioning.

**Method:**

736 Alzheimer's Disease Neuroimaging Initiative (ADNI) participants without dementia (341 Aβ+, 395 Aβ‐; 300 APOE ε4+, 436 APOE ε4‐) underwent neuropsychological testing including assessment of everyday functioning at baseline and annual follow‐up visits across two years. Florbetapir positron emission tomography (PET) imaging was used to measure Aβ deposition, and Aβ status (positive or negative) was determined based on established cutoffs. Everyday functioning was measured using the Functional Assessment Questionnaire (higher scores = greater difficulty). Linear mixed effects models adjusting for demographics, AD risk factors, and individual‐level mean cognitive performance, examined whether baseline IIV interacted with Aβ and APOE ε4 status on everyday functioning.

**Results:**

Higher baseline IIV predicted faster rate of decline in everyday functioning among Aβ+ relative to Aβ‐ participants (three‐way IIV x Aβ status x time interaction: b=0.13, 95% CI: [0.08, 0.17], *p* < .001) and among APOE ε4 carriers compared to non‐carriers (three‐way IIV x APOE ε4 status x time interaction: b=0.08, 95% CI: [0.03, 0.12], *p* < .001).

**Conclusion:**

Greater cognitive IIV was associated with faster functional decline, particularly among individuals who are Aβ+ or APOE ε4 carriers. Findings suggest that IIV may be a sensitive marker of brain changes and functional decline, offering added utility above and beyond conventional AD risk factors including age and gender as well as mean level of cognitive performance. Future research is needed to determine how to best operationalize IIV and establish reliability and validity of IIV in populations who better represent older adults who may be at greater risk for cognitive decline.

